# The Effects of Intravenous Iron Infusion on Preoperative Hemoglobin Concentration in Iron Deficiency Anemia: Retrospective Observational Study

**DOI:** 10.2196/31082

**Published:** 2022-02-03

**Authors:** Guy Nicholls, Rajan Mehta, Karen McVeagh, Matthew Egan

**Affiliations:** 1 Anaesthetics and Critical Care Southend University Hospital Southend on Sea United Kingdom; 2 East of England Ambulance Service Essex United Kingdom

**Keywords:** anemia, perioperative medicine, anesthetics, preoperative, perioperative, surgery, hemoglobin, hemoglobin concentration, iron deficiency, intravenous, blood

## Abstract

**Background:**

An iron infusion pathway using Ferrinject (ferric carboxymaltose) was implemented at Southend University Hospital for preoperative surgical patients with iron deficiency anemia undergoing major surgery. This was based on a treatment algorithm proposed by Munting and colleagues according to the international consensus statement on perioperative management of anemia and the UK National Institute for Health and Care Excellence (NICE) guidelines. These guidelines state that intravenous iron is indicated when oral iron is poorly tolerated or ineffective, there is insufficient time to surgery, or due to a functional iron deficiency.

**Objective:**

The objective of this study was to evaluate the change in adult hemoglobin (Hb) concentration (g/L) after Ferrinject infusion at the time of surgery.

**Methods:**

Data were retrospectively collected on all surgical patients that received an iron infusion preoperatively for iron deficiency anemia from July 2019 to April 2020. Nonsurgical, obstetric, and pediatric patients, and those without a postinfusion Hb level measurement were excluded. Data collected included the Hb, ferritin, and transferrin levels pre and postinfusion; correct dose of intravenous iron received; and any adverse reactions noted.

**Results:**

Thirty-two surgical patients with iron deficiency anemia received intravenous iron between July 2019 and April 2020 prior to surgery. The average pre and post iron infusion Hb concentration across the cohort was 97 g/L and 114 g/L, respectively (18% increase; *P*=.001). Two (6%) patients had a posttransfusion Hb level ≥130 g/L prior to surgery after infusion. Nine patients had both a pre and postinfusion ferritin level recorded, which showed an increase from 12 ng/mL preinfusion to 94 ng/mL postinfusion (*P*=.02). Twenty-three (72%) patients did not receive the full dose of intravenous iron based on their Hb level and weight. Twenty-four (75%) patients received an iron infusion >2 weeks prior to surgery and the other 8 (25%) patients received the infusion <2 weeks before their surgery. There was an average increase in Hb of 22% (21 g/L, 95% CI 13-28) and 5% (5 g/L, 95% CI 1-10), respectively, across the two groups (*P*=.03). There were no documented adverse reactions to intravenous iron.

**Conclusions:**

Intravenous iron is an effective intervention to improve the Hb concentration in patients with iron deficiency anemia despite the majority of patients not receiving the full dose based on their baseline Hb level and weight. Increasing the interval time between infusion and surgery was associated with a greater increase in Hb, with only a minimal increase observed if given less than 2 weeks prior to surgery.

## Introduction

Preoperative optimization is fundamental to improving surgical outcomes, with correction or improvement in iron deficiency anemia (IDA) being an important aspect in the preoperative management of patients undergoing major elective surgery [1]. This has resulted in a large drive across the United Kingdom for developing perioperative iron infusion pathways. Large studies have shown that approximately 40% of patients presenting for major surgery were anemic, with iron deficiency accounting for 80% of these cases [2].

We conducted a preliminary audit to determine the prevalence of IDA in patients undergoing major surgery at Southend University Hospital (Southend on Sea, UK) in 2018. We identified that 32% (52/187) of patients were anemic, when defined as a hemoglobin (Hb) level of <130 g/L for both men and women. Out of the 15 patients with available hematinics in the audit, 80% were shown to have IDA.

This preliminary audit in conjunction with the body of evidence of the benefits of correcting anemia prior to surgery led to the development of a local preoperative iron infusion pathway in 2019 for IDA. This was guided by the international consensus statement on the perioperative management of anemia and National Institute for Health and Care Excellence (NICE) guidelines [3,4]. We acknowledge that since the setting up of the pathway, the release of the results of the PREVENTT trial has raised a debate as to whether iron infusions to increase Hb levels improve clinical outcomes, although the average increase in Hb levels in the study was only 5 g/L [5,6].

In this local pathway, patients who were identified as having IDA during preoperative assessment were given an intravenous iron infusion in the form of Ferrinject (ferric carboxymaltose). The dose of Ferrinject, in grams, is based on weight and the starting Hb level (g/L). For an Hb level <100 g/L, the dose is 1.5 grams if the body weight is <70 kilograms and the dose is 2 grams if the body weight is >70 kilograms. If the Hb level is >100 g/L, the dose is 1 gram if the body weight is <70 kilograms and the dose is 1.5 grams if the body weight is >70 kilograms. A maximum dose of 1 gram can be given per infusion [6].

The objective of this study was to assess the impact of this formalized IDA preoperative pathway on the change in Hb concentration for patients with IDA undergoing major elective surgery.

## Methods

Adult surgical patients with IDA who received an intravenous iron infusion preoperatively through the pathway following its introduction in 2019 over a 9-month period were included in the study. Nonsurgical, obstetric, and pediatric patients were excluded. Patients were also excluded if there were no documented post iron infusion Hb levels.

Data were collected using electronic clinical records and laboratory results. Data collected included: patient demographics, pre and post iron infusion blood results (Hb, ferritin, and transferrin saturations), timing of infusion prior to surgery, whether the correct dose of Ferrinject was administered (as per the guideline), and if any adverse reactions to the infusion occurred. Preassessment proformas were assessed to determine if patients received oral iron in the preoperative period and the hospital’s blood bank checked to see if any blood transfusions were administered in the period leading up to surgery.

The primary objective was to assess the change in Hb concentration following an intravenous iron infusion of Ferrinject for patients with IDA undergoing major elective surgery. This was further analyzed to measure the change in Hb with respect to the length of time that the infusion was administered prior to surgery to assess the effects of earlier administration.

The secondary measures were the change in ferritin concentration postinfusion, proportion of patients who received the correct dose of intravenous iron (Ferrinject), proportion of patients with an Hb level >130 g/L at the time of surgery, and whether there were any adverse effects to the infusion.

Statistical analysis was performed using a paired Student *t* test following assessment for normality using the Kolmogorov-Smirnov test and the 95% CI. Statistical significance was defined as *P*<.05.

This study was approved by the hospital Trust’s Research and Development department. As no identifiable patient information was collected, patient consent was not required.

## Results

Thirty-five adult surgical patients received an intravenous iron infusion through the preoperative IDA pathway between July 2019 and April 2020. Three were excluded due to unavailable data. Baseline characteristics are summarized in Table 1. The median age was 65 years and the majority were men. The most common type of surgery performed by specialty was general surgery, followed by urology, gynecology, orthopedics, and combined general surgery/gynecology. There was no documentation of any patient receiving oral iron or a blood transfusion in the preoperative period.

**Table 1:** Baseline characteristics of the patients.

Characteristics			Value
Number of infusions, n			35
Cases excluded, n			3
Total cases analyzed, n			32
Age (years), mean (range)			65 (25-88)
Baseline Hb^a^ (g/L), mean			97
Male, n (%)			17 (53)
Pre and posttransfusion ferritin available, n			9
Preoperative oral iron, n			0
Preoperative blood transfusion, n			0
**Cases by specialty, n (%)**			
	General surgery	18 (56)	
	Gynecology	5 (16)	
	General surgery/gynecology combined	1 (3)	
	Urology	6 (19)	
	Orthopedics	2 (6)	

^a^Hb: hemoglobin.

For the primary outcome, the mean Hb level was increased from pre to post iron infusion by 18% on average for all cases. Significant increases were also found in the subanalyses for the <2 weeks group, 2 to 8 weeks group, and >8 weeks group. Overall, 75% of patients received an iron infusion >2 weeks prior to their surgery date, with an average increase in the Hb level of 22% compared to an increase of only 5% for those that received the iron infusion <2 weeks prior to surgery, representing a significant difference (Table 2).

With respect to the secondary outcomes (Table 3), preinfusion and postinfusion ferritin values were only available for 9 patients, demonstrating an overall mean increase. Three of the 32 patients exhibited a decrease in Hb at the time of surgery. Nine out of the 32 patients received the correct (full) dose of Ferrinject, and most of these patients were in the 2-8 weeks group, followed by the <2 weeks group and the >8 weeks group. Two patients had an Hb level of >130 g/L at the time of surgery post iron infusion, and there were no documented adverse effects to any of the infusions.

**Table 2:** Results for the primary outcome.

Cases	Preinfusion Hb^a^ (g/L)	Postinfusion Hb (g/L)	Change in Hb (95% CI)	*P* value
All cases	97	114	17 (11-23)	<.001
<2 weeks	103	108	5 (1-10)	.03
2-8 weeks	99	112	13 (2-24)	.03
>8 weeks	91	121	30 (21-39)	<.001
>2 weeks	95	116	21 (13-28)	<.001
>2 weeks vs >8 weeks	N/A^b^	N/A	N/A	.03

^a^Hb: hemoglobin.

^b^N/A: not applicable.

**Table 3:** Results for the secondary outcomes (N=32).

Outcome		Value
**Ferritin level (ng/mL), mean (95% CI)^a^**		
	Preinfusion	12 (1.3-22)
	Postinfusion	94 (32-156)
	Increase	82 (20-145)
**Timing of intravenous iron before surgery, n (%)**		
	<2 weeks	8 (25)
	2 to 8 weeks	13 (41)
	>8 weeks	11 (34)
	>2 weeks	24 (75)
**Patients receiving a full dose of intravenous iron, n (%)**		
	Overall	9 (28)
	<2 weeks	2 (25)
	2 to 8 weeks	5 (39)
	>8 weeks	2 (18)
Patients with a decrease in Hb^b^, n		3 (9)
Patients with Hb >130 g/L postinfusion, n (%)		2 (6)

^a^*P*=.02; data available for 9 patients.

^b^Hb: hemoglobin.

## Discussion

### Principal Findings

Although we were only able to identify 35 infusions over the 9-month study period, our results demonstrate that earlier administration of intravenous iron results in a far greater rise in Hb levels before surgery than when the infusion is given closer to the surgery date, in particular less than 2 weeks before surgery. Although this result appears to be intuitive, we felt it was important to assess our service to monitor the potential expected change in the Hb levels of our patients. When given less than 2 weeks before surgery, if only a 5 g/L rise is expected, then this is very unlikely to provide a benefit in the perioperative period compared to the 21 g/L rise found for the >2 weeks group, which has a much higher chance of providing early perioperative benefits and may also benefit the patient postoperatively.

As intravenous iron infusions have gained motion in becoming a cornerstone in patient blood management, the PREVENTT trial has raised important questions as to whether there are any improvements in clinical outcomes in treating anemia before surgery with intravenous iron. The study, published in October 2020 [5], showed no decrease in the need for blood transfusions or mortality compared to placebo, although the median time from infusion to surgery was only 15 days and only showed a small increase in Hb levels of 5 g/L. This may not be a sufficient increase to translate into a clinical benefit, particularly as studies into improving Hb levels preoperatively in other specialties have shown benefits, such as in orthopedics and cardiac surgery [7,8]. It is also important to note that patients with an Hb level of less than 90 g/L were excluded from this study and the outcomes were only available for major abdominal surgery.

Patients that receive their infusion less than 2 weeks before their surgery are most likely to be urgent surgical cancer patients. Although the PREVENTT trial may have shown no decrease in mortality or units of red cells transfused, these patients may benefit postoperatively from improved iron stores and the ability to increase their Hb level. One of the positive signals from PREVENTT was that improving Hb levels perioperatively may decrease the risk of being readmitted to hospital; however, further studies are needed to validate this finding. Moreover, if a larger rise in Hb is required to provide an early perioperative benefit, then patients awaiting elective surgery—such as in elective orthopedics where waiting times far exceed 8 weeks—may show a positive benefit, as evidenced by the increase in Hb of approximately 30 g/L for the group who received an infusion over 8 weeks before surgery.

There were three patients that showed a drop in Hb at the time of surgery. All of these patients were undergoing major cancer surgery (two were resections for bowel cancer and the other was a cystectomy); thus, we can only postulate as to whether this was due to failure of treatment, ongoing blood loss, or another underlying mechanism.

In our hospital, we have been using Ferrinject for our intravenous iron infusions, which has a maximal dose of 1 gram per infusion. Dosing is based on the starting Hb concentration and ideal body weight [9]. The majority of patients will require two infusions to receive the correct dose of intravenous iron with Ferrinject. Only a patient with a starting Hb >100 g/L and a body weight <70 kilograms can receive the full dose from one infusion as it would be 1 gram. A solution to this would be to switch to using ferric derisomaltose (Monofer) where a dose of up to 2 grams can be given per infusion [10]. Thus, switching to Monofer, in which the full intravenous iron dose can be given in a single administration, would provide cost savings to our service through not requiring two sessions to administer the full dose, thereby freeing up clinic time to provide additional treatments and easing the burden on patients in not having to come back for a second infusion. The raw cost of a vial of Ferrinject and Monofer is very similar. Calculations from the British National Formulary estimate a dose of 1, 1.5, and 2 grams costing £154.23 (≈US $230) vs £169.50 (≈US $230), £250 (≈US $338) vs £254 (≈US $344), and £309 (≈US $418) vs £339 (≈US $458), respectively [11].

Despite concerns raised regarding the safety profile of intravenous iron in the past, it was a positive finding that there no were documented adverse effects in any of the 32 infusions. Although our study is too small to make any meaningful conclusion on safety, a meta-analysis published in 2015 including 10,000 patients treated with different forms of iron replacement also found no increased risk of serious adverse effects with intravenous iron [12].

Out study does have several limitations, in particular the smaller than expected sample size after the preliminary audit. There is also the possibility of confounding factors; although we did attempt to remove these by checking if patients were taking oral iron or received a preoperative blood transfusion from the hospital records and prescriptions, we cannot completely rule out that they were not taking any iron supplementation that had not been prescribed, changed their dietary intake, or received a blood transfusion at another hospital.

### Conclusions

With this study, we believe that we have shown that intravenous iron is an effective treatment option for increasing Hb levels in IDA when given an appropriate timeframe to take effect and despite the majority of patients not receiving the full dose. An iron infusion should ideally be given greater than 2 weeks before surgery to achieve a clinically significant increase in the preoperative Hb concentration. At our hospital, a large proportion of surgical patients with IDA are not referred for intravenous iron and those that are tend to be underdosed when using Ferrinject. Our service will likely benefit from switching to Monofer or by improving pathways to allow patients to receive two infusions before their surgery.

Further study is required to fully quantify the rate of Hb increase and to determine whether increasing Hb levels preoperatively and increasing postoperative iron stores effectively decreases the number of blood transfusions required and improves postoperative outcomes.

## Abbreviations

Hb: hemoglobin
IDA: iron deficiency anemia
NICE: National Institute for Health and Care Excellence

## References

[1] Musallam K, Tamim H, Richards T, Spahn DR, Rosendaal FR, Habbal A, Khreiss M, Dahdaleh FS, Khavandi K, Sfeir PM, Soweid A, Hoballah JJ, Taher AT, Jamali FR (2011). Preoperative anaemia and postoperative outcomes in non-cardiac surgery: a retrospective cohort study. Lancet.

[2] Munting K, Klein AA (2019). Optimisation of pre-operative anaemia in patients before elective major surgery - why, who, when and how?. Anaesthesia.

[3] Muñoz M, Acheson A, Auerbach M, Besser M, Habler O, Kehlet H, Liumbruno GM, Lasocki S, Meybohm P, Rao Baikady R, Richards T, Shander A, So-Osman C, Spahn DR, Klein AA (2017). International consensus statement on the peri-operative management of anaemia and iron deficiency. Anaesthesia.

[4] (2015). Blood Transfusion NICE Guideline NG24.

[5] Richards T, Baikady R, Clevenger B, Butcher A, Abeysiri S, Chau M, Macdougall IC, Murphy G, Swinson R, Collier T, Van Dyck L, Browne J, Bradbury A, Dodd M, Evans R, Brealey D, Anker Sd, Klein A (2020). Preoperative intravenous iron to treat anaemia before major abdominal surgery (PREVENTT): a randomised, double-blind, controlled trial. Lancet.

[6] Vifor Pharma (2021). Ferinject (ferric carboxymaltose) - Summary of Product Characteristics. emc.

[7] Kotzé A, Carter L, Scally AJ (2012). Effect of a patient blood management programme on preoperative anaemia, transfusion rate, and outcome after primary hip or knee arthroplasty: a quality improvement cycle. Br J Anaesth.

[8] Rineau E, Chaudet A, Chassier C, Bizot P, Lasocki S (2016). Implementing a blood management protocol during the entire perioperative period allows a reduction in transfusion rate in major orthopedic surgery: a before-after study. Transfusion.

[9] Ferinject (ferric carboxymaltose) dosing and administration summary. Surrey and Sussex NHS.

[10] Pharmacosmos UK Ltd (2020). Monofer - Solution for injection/infusion - Summary of Product Characteristics. emc.

[11] (2021). Ferric Derisomaltose. British National Formulary.

[12] Avni T, Bieber A, Grossman A, Green H, Leibovici L, Gafter-Gvili A (2015). The safety of intravenous iron preparations: systematic review and meta-analysis. Mayo Clin Proc.

